# Hepatic Subcapsular Biloma: A Rare Complication of Laparoscopic Cholecystectomy

**DOI:** 10.1155/2014/186819

**Published:** 2014-08-10

**Authors:** Vassilios Stathopoulos, Marios Georganas, Konstantinos Stratakis, Eirini Delaporta, Emmanouil Karallas, Konstantinos Koutsopoulos

**Affiliations:** ^1^2nd Department of Surgery, Rhodes General Hospital, 85100 Rhodes, Greece; ^2^Department of Interventional Radiology, Rhodes General Hospital, 85100 Rhodes, Greece; ^3^Department of Radiology, Rhodes General Hospital, 85100 Rhodes, Greece

## Abstract

The development of an intra-abdominal bile collection (biloma) is an infrequent complication of laparoscopic cholecystectomy (LC). These bilomas develop in the subhepatic space most often secondary to iatrogenic injury of the extrahepatic ducts. We present a case of hepatic subcapsular biloma following LC and we discuss its etiology and management. Early diagnosis is crucial and percutaneous drainage under CT guidance should be employed to resolve this complication.

## 1. Introduction

Laparoscopic cholecystectomy (LC) is the treatment of choice for the management of symptomatic gallstones [1]. The first procedure was performed in 1987 by Mouret. Since then various complications have been reported [2–5]. A PubMed/Medline literature search using the mesh term “hepatic subcapsular biloma” resulted in only 14 items. Such bilomas are thus rare complications of LC. We describe the case of a 65-year-old male who developed a hepatic subcapsular biloma following LC.

## 2. Case Report

A 65-year-old male was admitted for elective LC. He had a history of right upper quadrant pain and fatty food intolerance. An ultrasound examination of the abdomen revealed a single gallstone in the gallbladder which was 0.6 cm in diameter, no hepatic abnormalities, and a normal common bile duct of 0.4 cm in diameter. Physical examination and preoperative blood tests were within normal limits.

A LC was performed under general anesthesia. The procedure was difficult due to adhesions between the cystic duct, the omentum, and the duodenum, while the gallbladder itself was sclerotic and hard to manipulate. Once the junction between the cystic and the common bile duct was identified laparoscopically, we decided not to perform an intraoperative cholangiogram and continue the LC without any incidents. After the completion of the LC, there was no bile-stained fluid present after a meticulous lavage of the peritoneal cavity with 0.9% normal saline solution.

**Figure 1:**
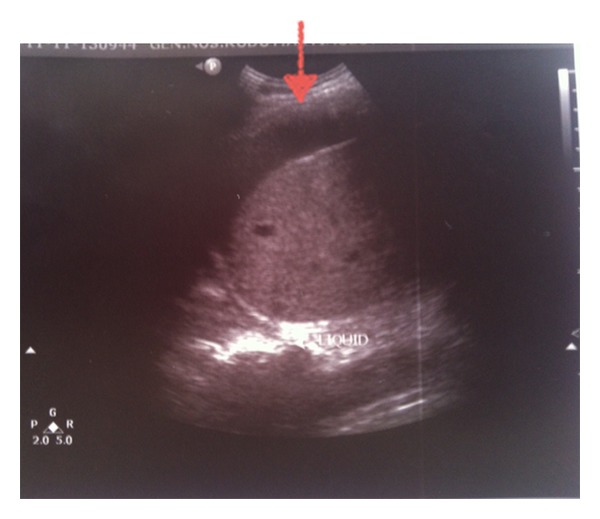
Ultrasound examination of the abdomen showing a hypoechoic subcapsular collection of the liver (arrow).

**Figure 2:**
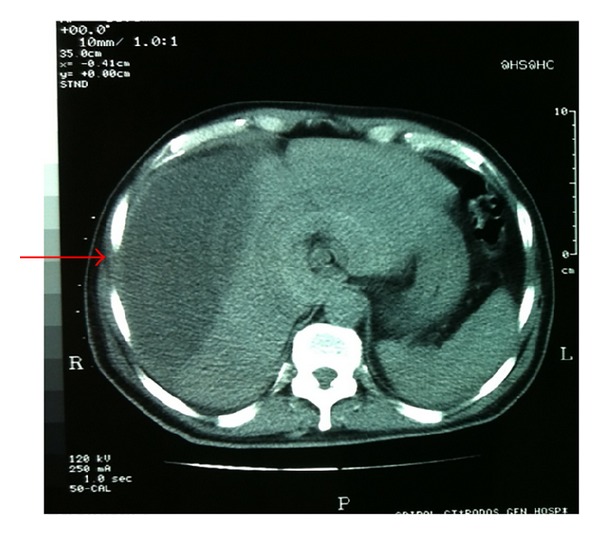
CT of the abdomen showing the subcapsular collection (arrow).

**Figure 3:**
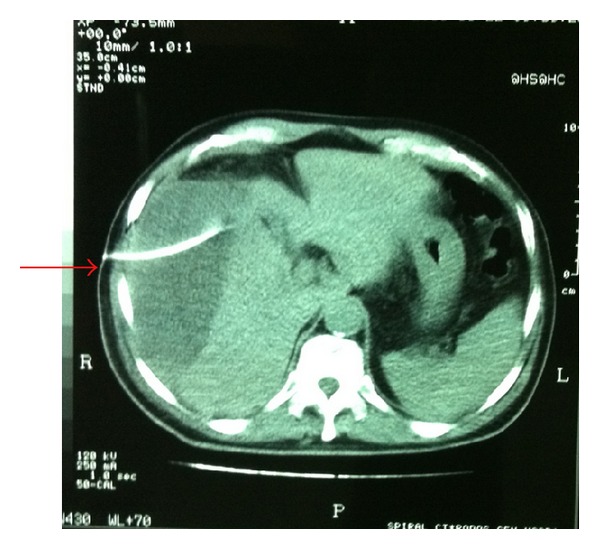
Percutaneous drainage of the collection under CT guidance (arrow).

**Figure 4:**
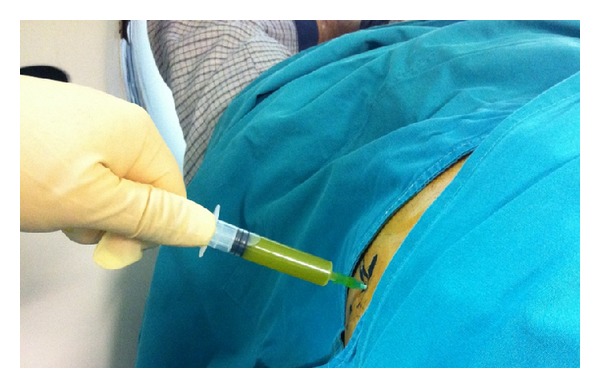
Bile-stained fluid drawn percutaneously.

**Figure 5:**
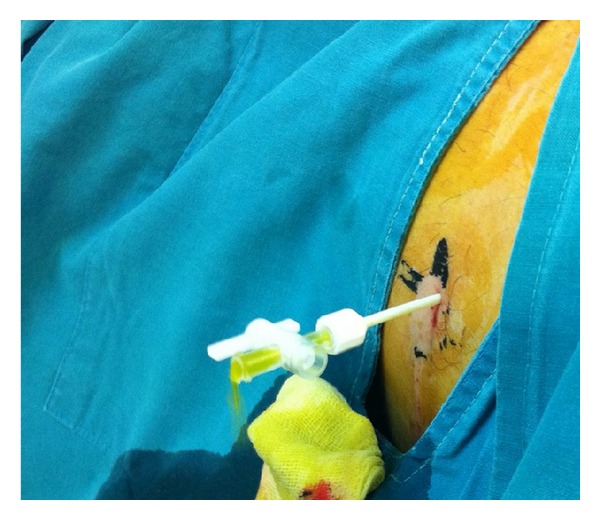
Bile-stained fluid drawn by the catheter upon insertion.

A Penrose drain was left in the subhepatic space following the procedure. A total of 70 cc of bile stained fluid were drained during the first 24 hours postoperatively. On the subsequent 6 days, a total of 440, 400, 350, 210, 100, and 50 cc of bile, respectively, were drained. The patient was discharged in good condition, with the drain left in place. During his hospitalization the patient had no fever and no elevated white blood cell count or liver enzymes, and the postoperative U/S of the abdomen revealed no intra-abdominal fluid collection. On the 16th postoperative day, he was readmitted to hospital with severe right upper quadrant and right subcostal pain and chills. The patient had a white blood cell count of 16,000 cells/*μ*L but no fever upon readmission. A subhepatic fluid collection was suspected. An ultrasound examination of the abdomen revealed no significant collection in the subhepatic space but a large hypoechoic subcapsular collection in the right lobe of the liver (Figure 1). The patient had metal prosthesis and metallic parts in his body as a result of an orthopaedic surgery 20 years ago, so we could not perform an MRI or MRCP and a CT scan was then performed. The density of the subcapsular collection (Figure 2) did not suggest the presence of blood, so it was decided to perform a percutaneous drainage of the collection under CT guidance (Figure 3). An 8-Fr pigtail locking loop catheter (Boston Scientific, USA) was inserted in the cavity under local anesthesia and CT guidance using the Seldinger technique. This pigtail drained 1300 cc of bile-stained fluid on insertion (Figures 4 and 5). On the following 2 days, only 50 cc and 10 cc were drained. The patient remained in hospital for another 7 days until his white blood cell count became normal and no further bile-stained fluid was drained by the pigtail catheter. The catheter was then removed. An abdominal ultrasound 1 week later was normal.

## 3. Literature Review and Discussion

The term biloma was first introduced by Gould and Patel in 1979 to describe a well-differentiated collection of bile outside the biliary tree [6]. Kuligowska et al. extended the term to include also intrahepatic collection of bile [7]. Although bile collection in the peritoneal cavity is a well-described complication after open or LC [2–5], the hepatic subcapsular biloma is a rare complication [8, 9].

In our case the diagnosis was established by ultrasound followed by an abdominal CT scan, and the complication was resolved with a percutaneous evacuation of the biloma under CT guidance. The draining catheter was placed under CT guidance to avoid any further complications, such as biloma of the subpleural cavity. On the other hand the size and the type of the catheter were decided once the first sample of the collection was obtained. We decided to use an 8-Fr pigtail locking loop catheter (Boston Scientific, USA) and not a 10-Fr one which is slightly more traumatic. We also selected a locking loop catheter because it is more difficult to be removed accidentally. The Seldinger technique is a well-established procedure in clinical practice used to introduce catheters, with predilatation of the initial track in addition before placing the final drainage. This technique is less traumatic and more detailed than the one-step Trocar method.

Some authors attribute the development of a biloma to a small biliary branch due to the backpressure associated with the high-pressure irrigation used during intraoperative cholangiogram [10]. However, a cholangiogram was not performed in our patient. We think that the possible etiology for the subcapsular biloma in this patient was a disruption of a small biliary branch near the gallbladder bed during dissection because the procedure was technically difficult. The fact that the Penrose drain of the subhepatic space yielded bile-stained fluid strengthens the hypothesis of an iatrogenic injury of a small peripheral biliary branch (possibly with electrocauterisation) and the resulting bile leakage into the subcapsular space. The distension of Glisson's capsule caused the right upper quadrant abdominal pain [10]. We suggest that placing a drainage in the subhepatic space should be used in all cases of LC, due to the fact that biliary peritonitis is a life-threatening situation with occasionally minimum clinical and laboratory findings. Routine cholangiography during LC is still debatable [11–15]. In our institute we reserve this procedure only for patients with unclear anatomical route of the extrahepatic bile ducts. Instead, we perform a triple diagnostic test preoperatively including medical history, routine liver function tests, and ultrasound examination of the right upper quadrant of the abdomen [16].

In conclusion, a subcapsular biloma is a rare complication of LC. Early diagnosis and percutaneous drainage under CT guidance is the key to resolve this complication.

## References

[1] (1994). *Second International Congress of the European Association for Endoscopic Surgery Consensus Conference: Cholecystectomy. Madrid, Spain, September 1994*.

[2] Deyo GA (1992). Complications of laparoscopic cholecystectomy. *Surgical Laparoscopy and Endoscopy*.

[3] Ferguson CM, Rattner DW, Warshaw AL (1992). Bile duct injury in laparoscopic cholecystectomy. *Surgical Laparoscopy and Endoscopy*.

[4] Wolfe BM, Gardiner BN, Leary BF, Frey CF (1991). Endoscopic cholecystectomy: an analysis of complications. *Archives of Surgery*.

[5] Huang X, Feng Y, Huang Z (1997). Complications of laparoscopic cholecystectomy in China: an analysis of 39 238 cases. *Chinese Medical Journal*.

[6] Gould L, Patel A (1979). Ultrasound detection of extrahepatic encapsulated bile: “biloma”. *American Journal of Roentgenology*.

[7] Kuligowska E, Schlesinger A, Miller KB, Lee VW, Grosso D (1983). Bilomas: a new approach to the diagnosis and treatment. *Gastrointestinal Radiology*.

[8] Hassani KIM, Benjelloun EB, Ousadden A, Mazaz K, Taleb KA (2009). A rare case of hepatic sub capsular biloma after open cholecystectomy: a case report. *Cases Journal*.

[9] Vazquez JL, Thorsen MK, Dodds WJ (1985). Evaluation and treatment of intraabdominal bilomas. *The American Journal of Roentgenology*.

[10] Braithwaite BM, Cabanilla LT, Lilly M (2003). Hepatic subcapsular biloma: a rare complication of laparoscopic cholecystectomy and common bile duct exploration.. *Current surgery*.

[11] Richardson MC, Bell G, Fullarton GM (1996). Incidence and nature of bile duct injuries following laparoscopic cholecystectomy: an audit of 5913 cases. West of Scotland Laparoscopic Cholecystectomy Audit Group. *British Journal of Surgery*.

[12] Snow LL, Weinstein LS, Hannon JK, Lane DR (2001). Evaluation of operative cholangiography in 2043 patients undergoing laparoscopic cholecystectomy: a case for the selective operative cholangiogram. *Surgical Endoscopy*.

[13] Metcalfe MS, Ong T, Bruening MH, Iswariah H, Wemyss-Holden SA, Maddern GJ (2004). Is laparoscopic intraoperative cholangiogram a matter of routine?. *The American Journal of Surgery*.

[14] Lepner U, Grünthal V (2005). Intraoperative cholangiography can be safely omitted during laparoscopic cholecystectomy: a prospective study of 413 consecutive patients. *Scandinavian Journal of Surgery*.

[15] Akolekar D, Nixon SJ, Parks RW (2009). Intraoperative cholangiography in modern surgical practice. *Digestive Surgery*.

[16] Pourseidi B, Khorram-Manesh A (2007). Triple non-invasive diagnostic test for exclusion of common bile ducts stones before laparoscopic cholecystectomy. *World Journal of Gastroenterology*.

